# Clinical Reclassification of Vitamin D Status Across Three Automated Immunoassays After Comparability Study

**DOI:** 10.3390/nu18142267

**Published:** 2026-07-10

**Authors:** Adina Huțanu, Ana-Maria Fotache (Țurcan), Oana Roxana Oprea, Andreea Truța, Andrea Márta Fodor, Minodora Dobreanu

**Affiliations:** 1 Department of Laboratory Medicine, George Emil Palade University of Medicine, Pharmacy, Science, and Technology of Targu Mures, 540142 Târgu Mureș, Romania; adina.hutanu@umfst.ro (A.H.); andrea.fodor@umfst.ro (A.M.F.); minodora.dobreanu@umfst.ro (M.D.); 2 Advanced Medical and Pharmaceutical Research Center, George Emil Palade University of Medicine, Pharmacy, Science, and Technology of Targu Mureș, 540139 Târgu Mureș, Romania; 3 Doctoral School of Medicine and Pharmacy (I.O.S.U.D.), George Emil Palade University of Medicine, Pharmacy, Science, and Technology of Târgu Mure, 540139 Târgu Mureș, Romania; anamaria.turcan@umfst.ro; 4 Central Laboratory, Emergency Clinical County Hospital Targu Mureș, 540136 Târgu Mureș, Romania; trutaandreea1@yahoo.com

**Keywords:** 25(OH) vitamin D, clinical decision threshold, method comparison, reclassification rate

## Abstract

**Background/Objectives**: Variability between routine laboratory immunoassays for total 25-hydroxyvitamin D (25(OH) vitamin D) measurement may impact clinical interpretation, particularly around clinical decision thresholds. The aim of the study was to evaluate the analytical comparability and clinical interchangeability between three automated immunoassay platforms using chemiluminescent assays (CLIA) and electrochemiluminescent assay (ECLIA) principles for total 25(OH) vitamin D and to evaluate the assay-dependent impact on patient reclassification. **Methods**: A total of 83 serum samples were analyzed using three automated immunoassay platforms and the between method comparability was assessed. Additionally, clinical agreement and reclassification rate were evaluated at two clinical decision thresholds, 20 ng/mL for insufficiency and 12 ng/mL for severe deficiency. **Results**: The comparability analysis revealed a systematic difference between methods, with a high reclassification rate between ECLIA compared to CLIA methods. Additionally, the expanded measurement uncertainty generates a clinically relevant gray zone around the clinically relevant thresholds. **Conclusions**: Routinely used immunoassays for total 25(OH) vitamin D measurement in clinical laboratories are not fully interchangeable, furthermore the measurement uncertainty contributes to patient reclassification, especially around the threshold of 20 ng/mL.

## 1. Introduction

Vitamin D is an essential parameter for calcium homeostasis and bone metabolism, also involved in different pathologies, including cardiovascular disease [1], cancers [2] and immune dysregulation [3]. Although in many cases the supplementation failed to prove significant positive effects, the genetic evidence suggests a link between vitamin D status and autoimmune diseases, with clinical implications and disease prevention [4]. The assessment of vitamin D status is achieved by measuring plasma or serum levels of total 25(OH) vitamin D, the most reliable indicator, which consists of the sum of 25(OH) vitamin D_2_ and 25(OH) vitamin D_3_. Over the last few years, the laboratories have faced an enormous increase in the measurement of total vitamin D, based on the assumption of the widespread deficiency in this micronutrient. However, the accurate measurement of the total 25(OH) vitamin D remains a challenge for the laboratories, as the Vitamin D Standardization Program (VDSP) revealed [5]. Several methods have been developed for measuring total 25(OH) vitamin D, most of which employ immunoassays. However, the preferred method is Isotope Dilution Liquid Chromatography-Tandem Mass Spectrometry (ID LC-MS/MS) using the reference materials already available from the National Institute of Standards and Technology (NIST), containing vitamin D metabolites in various concentrations [6]. Most methods are traceable to the reference materials provided by NIST, either serum matrix Standard Reference Material (SRM) or an ethanolic solution of 25(OH)vitamin D_2_, 25(OH)vitamin D_3_, and metabolites with known concentrations Certified Reference Materials (CRMs) [7]. Apart from the reference materials, the differences between methods for measuring vitamin D include the variability derived from vitamin D binding protein, as the release of vitamin D from its carrier protein is crucial for an accurate measurement. Furthermore, apart from vitamin D binding protein (VDBP), 25(OH) vitamin D is bound to other transport proteins, including albumin and lipoproteins, whose concentrations and binding affinities vary with physiological conditions such as pregnancy and renal disease. Moreover, the type of assay used plays a significant role in the measurement outcomes. While LC-MS/MS methods are generally less susceptible to interference from endogenous factors, these methods remain vulnerable to inaccuracies generated by inadequate standardized calibration, inadequate use of the internal standards, quality control or matrix effect. On the other hand, immunoassays can encounter numerous potential interferences that may affect the results [7,8]. The accurate quantification remains challenging due to the presence of multiple biologically active forms, the lipophilic nature of the molecule, and interference from metabolic byproducts and epimers [9]. These factors introduce additional variability in measurement.

Another practical issue in the interpretation of vitamin D levels is the inconsistency in defining the clinical thresholds; while the 2011 guideline established a cut-off at 20 ng/mL for deficiency and above ≥30 ng/mL for optimal levels [10], these thresholds were revised in 2025, suggesting that only marked deficiency, defined as total 25(OH) vitamin D levels ≤12 ng/mL, should be taken into account [11]. However, in Romania, the guidelines for the evaluation of the vitamin D status and supplementation recommends the threshold of 12 ng/mL for deficiency while values between 12 and 20 ng/mL were considered insufficient [12]. Established reference intervals and clinical decision cut-off values are particularly important given the high prevalence of vitamin D deficiency among both inpatient and outpatient populations referred for laboratory testing, with a reported prevalence of approximately 70% in the same Romanian region as our study population [13].

Although the LC-MS/MS is considered the best method for assessing total 25(OH) vitamin D status, immunoassays are most suitable for routine clinical laboratories, mostly because of their high throughput and shortened turnaround time. Despite the development of reference materials and manufacturers’ claims of traceability and good agreement with established reference methods, significant variability remains among different immunoassay techniques. This inconsistency highlights the complexity of accurately measuring total 25(OH) vitamin D levels, underscoring the need for further evaluation of these methods. In clinical practice, it is crucial to acknowledge these discrepancies, as they can significantly influence the interpretation of patients’ vitamin D status.

The objective of the study was to evaluate total 25(OH) vitamin D immunoassays across three different automated analyzers, utilizing two analytical principles (CLIA and ECLIA), and to compare their performance. The second objective was to evaluate how the biases between methods will affect the classification of the patients into specific categories, particularly in distinguishing between deficiency and sufficiency.

## 2. Materials and Methods

### 2.1. Samples Characteristics

For the comparability study, leftover samples of healthy individuals routinely investigated for various routine parameters and for total 25(OH) vitamin D status, were used. Blood collection was performed in a fasting state, in serum separator tubes (SST), maintained at room temperature for 30 min for clot formation before centrifugation for 15 min at 1500× *g*, at room temperature. The samples were processed following good laboratory practice and tested first on ECLIA platform in line with the laboratory’s routine testing schedule, according to the manufacturer’s specification. For the CLIA measurement, leftover serum samples were aliquoted in two separate cryovials and stored at −80 °C until analysis was completed, without multiple freeze–thaw cycles (analyte stability 3 months at −80 °C). The study was approved by the institutional Ethics Committee (30032/05.12.2022) and informed consent was waived. The study was conducted in accordance with the Clinical and Laboratory Standards Institute (CLSI) EPI09-A3 guideline for comparability between quantitative methods using patient samples (https://clsi.org (accessed 7 May 2026)) [14]. Samples were analyzed in the laboratory for total 25(OH) vitamin D status using the ECLIA method with the Cobas e801 analyzer (Roche Diagnostics, Mannheim, Germany) (*n* = 81), and with two additional instruments, Liaison XL (*n* = 82) (DiaSorin, Italy) and Maglumi X6 (SNIBE, Shenzhen, China) (*n* = 82), both using CLIA technology. Because of occasional sample volume limitations or analytical exclusions, the number of valid measurements slightly differed between platforms. The measurement of the total 25(OH) vitamin D was performed according to each manufacturer’s instructions, following good laboratory practice.

### 2.2. Methods for Total 25(OH)Vitamin D Measurement

#### 2.2.1. 25(OH) Vitamin D Measured by ECLIA on Cobas e801

The Elecsys Vitamin D Total III assay (REF:09038086190) performed on the Cobas e801 platform (Roche Diagnostics, Mannheim, Germany) is an electrochemiluminescence binding assay (ECLIA) with a competitive principle. Samples are pretreated to release vitamin D from its binding protein (VDBP). The method is traceable to NIST SRM 2972 reference material. The manufacturer declares an analytical measurement range (AMR) of 3.0–120 ng/mL, with a limit of detection (LoD) of 3.0 ng/mL and a limit of quantitation (LoQ) of 6 ng/mL. 25(OH) vitamin D is released from the endogenous VDBP during the first incubation, when 9 µL of sample is pretreated with reagent 1 (Ditiotreitol 1) and reagent 2 (sodium hydroxide 57.5 g/L). During the second incubation, the pretreated samples react with ruthenium-labeled vitamin D binding protein, resulting in the labeled reaction immune complexes. The manufacturer reports in the instructions for use (IFU) a 100% cross-reactivity with 3-epi-25(OH) vitamin D_2_ and D_3_, and <10% for 1,25-dihydroxyvitamin D_2_, 1,25-dihydroxyvitamin D_3_, vitamin D_2_, and vitamin D_3_ (https://elabdoc-prod.roche.com/eLD/web/ro/ro/home (accessed on 7 May 2026)). Also, the manufacturer declared 100% reactivity with 25(OH) vitamin D_2_ and 25(OH) vitamin D_3_ [15]. The manufacturer’s performance characteristics in the IFU are an inter-run CVs of 4.7% for a mean concentration of 20.2 ng/mL, and 2.9% for a mean concentration of 38.2 ng/mL, based on internal quality control level 1 and 2.

#### 2.2.2. 25(OH) Vitamin D Measured by CLIA Method on Liaison XL

The measurement of total 25(OH) vitamin D on the Liaison XL (DiaSorin, Italy) platform uses a chemiluminescence immunoassay (CLIA) based on the competitive binding principle, reagent LIAISON 25 OH Vitamin D Total Assay (REF:310600). The method is traceable to NIST SRM 2972, and the AMR reported by the manufacturer is 4–150 ng/mL, with a LoD of 3.8 ng/mL. The 25(OH) vitamin D from the samples is released from the VDBP during the first incubation, afterwards binding to a specific anti 25(OH) vitamin D antibody captured on the solid phase of the reaction. The manufacturer reports cross-reactivity with metabolites such as 1,25-dihydroxy vitamin D_2_ and D_3_, 3-epi-25-hydroxy vitamin D_3_, and vitamin D_2_ and D_3_ to be lower than 10%, with 100% cross-reactivity with 25(OH) vitamin D_2_ and 25(OH) vitamin D_3_. The performance characteristics declared in the IFU for inter-run estimation based on internal laboratory QC were CV of 8.9% at a mean concentration of 17.2 ng/mL, and a CV of 6.0% at a mean concentration of 52 ng/mL.

#### 2.2.3. 25(OH) Vitamin D Measured by CLIA Method on Maglumi X6

The measurement of the total 25(OH) vitamin D (CLIA) with Maglumi 25(OH) vitamin D (REF:1320261004M) on Maglumi X6 involves a sandwich immunoassay for small molecules, where magnetic beads coated with specific antibodies interact with the 25(OH) vitamin D_2_ + vitamin D_3_ from the sample forming immune complexes, after pretreatment with a releasing agent for dissociation from VDBP. The method is traceable against the NIST SRM 2972a reference material. The AMR declared in the IFU for 25(OH)vitamin D is 1.50–150 ng/mL, with the LoD of 0.5 ng/mL and LoQ of 1.5 ng/mL. The cross-reactivity for Maglumi 25(OH)vitamin D was tested for three metabolites: 1,25-dihydroxy-vitamin D_2_; 1,25-dihydroxy-vitamin D_3_; and 3-epi 25-OH vitamin D_3_, with no interferences observed up to 100 ng/mL; however, the manufacturer did not provide a separate percentage for reactivity to 25(OH) vitamin D_2_ and 25(OH) vitamin D_3_. Other metabolites showed a very low cross-reactivity (<0.5%) according to the manufacturer’s claim. The inter-run imprecision based on quality control material, according to IFU was 6.48% for a mean concentration of 20 ng/mL and CV of 4.57% at a mean concentration of 50 ng/mL.

### 2.3. Statistical Analysis

Method comparison was performed with Bland–Altman analysis, estimated as absolute concentration and percentage of differences, with 95% limits of agreement. The analysis was performed over the entire measurement interval and within the clinically relevant decision range (10–30 ng/mL). The Spearman rank coefficient and Passing–Bablok regression were used for the evaluation of the correlation between the methods (each immunoassay against the other). Furthermore, the samples were categorized based on key clinical decision thresholds: ≥20 ng/mL (sufficient) and <12 ng/mL (severe deficiency), allowing for patient reclassification according to these criteria, and evaluating the clinical impact of the inter-method variability. The samples distribution across categories was compared between the three immune methods, assessing the proportion of the reclassified subjects. Pairwise agreement between methods was assessed using Cohen’s kappa coefficient.

Statistical analysis was performed with MedCalc^®^Statistical Software v20.104 (MedCalc Software Ltd., Ostend, Belgium; https://www.medcalc.org, (accessed on 7 May 2026)).

### 2.4. Bias Estimation

Ten samples from the Vitamin D External Quality Assessment Scheme (DEQAS) (London, UK), five samples/run in two consecutive runs, were used for bias estimation. The values for 25(OH) vitamin D_3_ and 25(OH) vitamin D_2_ were assigned by the Center for Disease Control (CDC) Reference Measurement Procedure while the values for 3-epi-25-(OH) vitamin D_3_ and 24,25-dihydroxy-vitaminD_3_ (24,25(OH)_2_ vitamin D_3_) were assigned by the CDC with an LC-MS/MS method. The DEQAS proficiency testing samples were measured on two consecutive occasions: October 2025 and January 2026. The external proficiency results obtained from DEQAS were used for each platform for bias estimation (absolute and percentage) in relation to target value assigned by LC-MS/MS method (CDC traceable) and against the laboratory trimmed mean (ALTM). For the interpretation of analytical performance, the mean bias was evaluated against standardization-based performance goals, particularly those proposed by the Vitamin D Standardization Program (VDSP) [16].

The measurement uncertainty was estimated using the imprecision derived from the internal quality control data and the uncertainty of the calibrators obtained from manufacturers traceability certificate. Expanded uncertainty (U) was calculated using a coverage factor k = 2. The imprecision data were obtained from internal quality control measurements using two levels of QC material for each instrument. Total error (TE) of the methods was calculated as |Bias| + 1.65 × CV.

## 3. Results

### 3.1. Sample Characteristics

Sample distribution across clinically relevant thresholds is presented in Table 1. The proportion of severe deficiency (<12 ng/mL) showed minor variation between platforms (4.8% for Liaison XL to 7.3% for Maglumi X6). In contrast, the proportion of samples classified as sufficient (≥20 ng/mL) varied substantially, ranging from 54.3% for Liaison XL to 70.7% for Maglumi X6. Median vitamin D concentrations also differed between methods, with higher values observed for Liaison XL and Maglumi X6 compared to Cobas e801.

### 3.2. Agreement in Classification at the Established Clinical Decision Levels of 20 ng/mL

Pairwise agreement between immunoassays is presented in Table 2. At the 20 ng/mL threshold, Cohen’s kappa indicated moderate to very good agreement, ranging from 0.642 (Cobas vs. Liaison XL) to 0.913 (Liaison XL vs. Maglumi X6).

**Figure 1:**
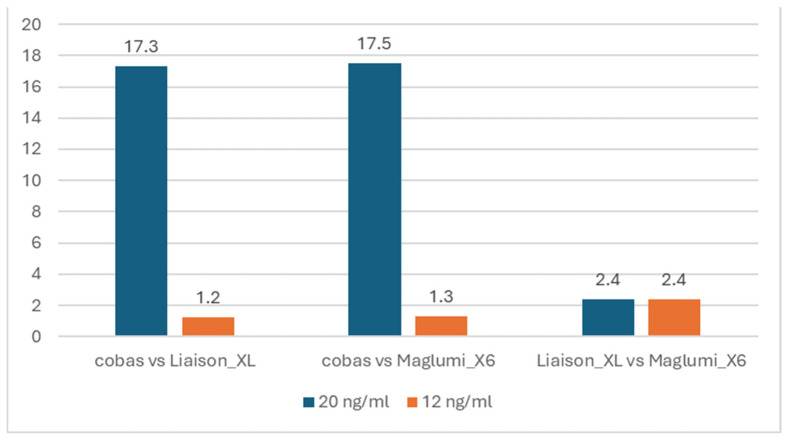
Assay-dependent classification rate at clinical decision levels (<20 ng/mL and <12 ng/mL) for all three platforms. The graph represents the proportion of samples classified discordantly between ECLIA and CLIA instruments.

At the 12 ng/mL threshold, agreement was perfect across all comparisons (κ = 1.00). Despite this, a substantial proportion of samples were classified depending on the analytical platform, particularly at the 20 ng/mL threshold. Cobas e801 systematically classified more samples below the sufficiency threshold, with reclassification rates of 17.3% and 17.5% when compared to Liaison XL and Maglumi X6, respectively. This discordant classification was predominantly unidirectional, with Cobas reporting lower concentrations. In contrast, agreement between the two CLIA-based methods (Liaison XL and Maglumi X6) was markedly higher, with minimal discordance in classification (2.4%). The percentages represent assay-dependent patients’ classification across the evaluated immunoassays, defined as samples categorized on the opposite side of the cut-off, depending on the instruments. As none of the assays used was considered a reference method for the vitamin D measurement, the results reflect inter-method classification disagreement rather than diagnostic misclassification. However, at the severe deficiency threshold (12 ng/mL), the differences were negligible (≤2.4%), indicating high concordance at low concentration levels (Figure 1).

### 3.3. Agreement in Classification at the Established Clinical Decision Levels

#### 3.3.1. Passing–Bablok Regression Analysis

**Figure 2:**
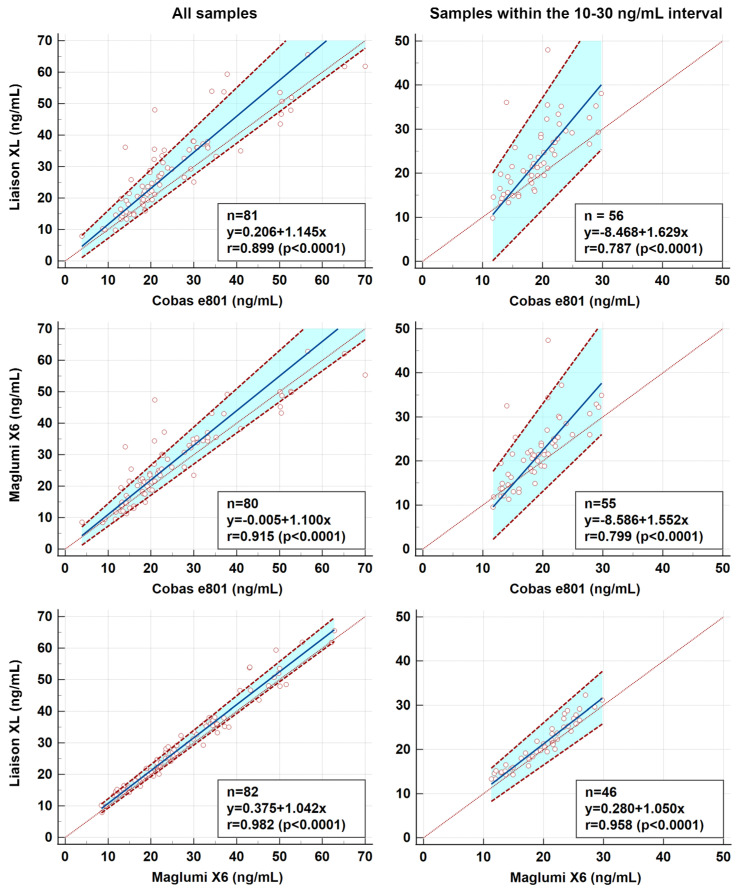
Passing–Bablok regression analysis of paired samples for the entire dataset (**left**) and for samples within the clinical decision interval of 10–30 ng/mL (**right**). The clinical decision interval was defined based on the results obtained with the analyzer displayed on the horizontal (X) axis.

Passing–Bablok regression analysis demonstrated strong correlations between methods across the entire analytical range (Table 3), with Spearman coefficients ranging from 0.899 to 0.982. A proportional bias was observed for comparisons involving Cobas e801, particularly within the clinical decision interval (10–30 ng/mL), where slopes exceeded 1.5 when compared with Liaison XL and Maglumi X6 (Figure 2).

In contrast, the agreement between Liaison XL and Maglumi X6 remained close to identity (slope 1.042–1.099), indicating minimal proportional bias. No significant deviation from linearity was observed for most method comparisons, except for a borderline deviation between Cobas and Maglumi X6 (*p* = 0.05).

#### 3.3.2. Bland–Altman Analysis

**Figure 3:**
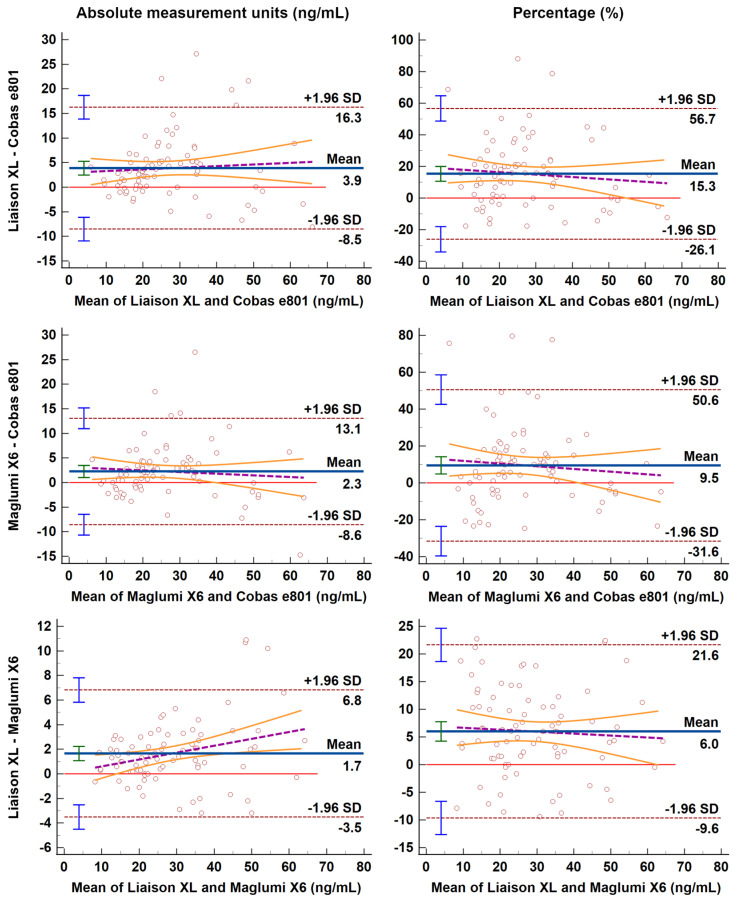
Paired Bland–Altman analysis between the three instruments for all samples, expressed in absolute measurement units (ng/mL, (**left**)) and percentage values (%, (**right**)).

**Figure 4:**
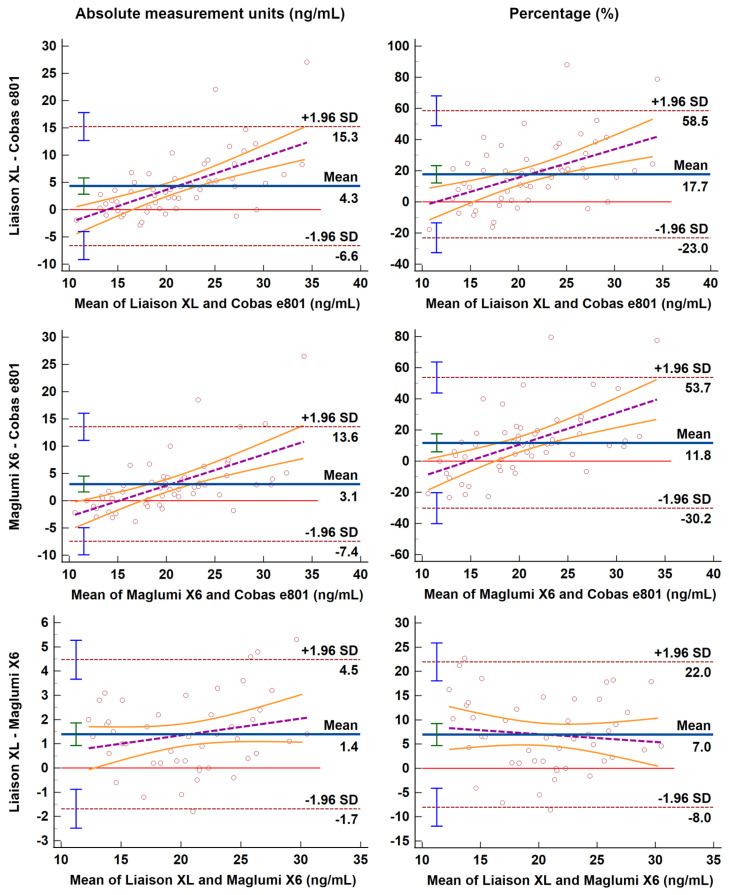
Paired Bland–Altman analysis between the three instruments for samples within the 10–30 ng/mL interval, expressed in absolute measurement units (ng/mL, (**left**)) and percentage values (%, (**right**)).

Bland–Altman analysis confirmed a systematic positive bias of CLIA-based methods (Liaison and Maglumi) compared to Cobas e801. For Liaison XL vs. Cobas, the mean difference was 3.9 ng/mL (15.3%), with wide limits of agreement, particularly within the clinical decision interval, where the bias was slightly higher in both measurement units (4.3 ng/mL) and percentage (17.7%). Similarly, Maglumi X6 vs. Cobas showed a mean difference of 2.3 ng/mL (9.5%), with a similarly slightly higher bias within the clinical decision range: 3.1 ng/mL, that is 11.8%. In contrast, agreement between Liaison XL and Maglumi X6 was markedly better, with similar bias in both the global analysis (1.7 ng/mL, 6.0%) and the clinical decision interval (1.4 ng/mL, 7.0%), as well as narrower limits of agreement. Overall, the magnitude of disagreement was greatest for comparisons involving Cobas e801 and most pronounced within the clinical decision interval (Figure 3 and Figure 4).

### 3.4. Results from DEQAS External Proficiency Testing

**Figure 5:**
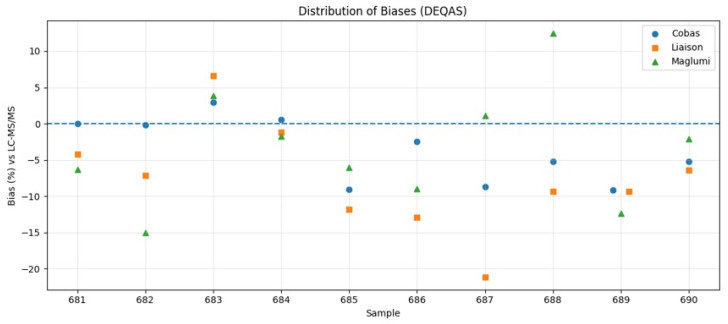
The target values of CDC DEQAS samples were: 48.0 nmol/L, 60.6 nmol/L, 76.3 nmol/L, 92.4 nmol/L, 142.3 nmol/L, 43.4 nmol/L, 43.8 nmol/L, 68.9 nmol/L, 81.6 nmol/L and 42.2 nmol/L (the conversion formula for non-chromatographic assays for conversion from ng/mL to nmol/L provided by the DEQAS was: Total 25(OH) vitamin D ng/mL × 2.5). The figure shows the individual biases for each DEQAS samples for all three analyzers. Sample (685) was enriched with 25(OH) vitamin D_2_.

The results from the DEQAS external quality assessment scheme are summarized in Table 4. Mean bias relative to the CDC values ranged from −7.6% (Liaison XL) to −3.6% (Cobas e801 and Maglumi X6). Bias relative to ALTM was minimal for Cobas and Maglumi but remained negative for Liaison XL. A specific DEQAS sample (685) was enriched with 25(OH) vitamin D_2_ (Figure 5) resulting in substantial deviation from LC-MS/MS assigned values, consistent with findings reported in other studies. The mean biases for all three analyzers derived from DEQAS external Quality Program are summarized in Table 4, while the individual biases from assigned CDC target values are depicted in Figure 5.

### 3.5. Measurement Uncertainty and TE Estimation

Measurement uncertainty, bias and total error estimates for all three platforms are presented in Table 5, using data derived from internal quality controls level 1 and level 2 (coefficients of variation), and calibrators uncertainty provided by the manufacturers. Internal QC was performed on each analyzers using two levels of controls, level 1 representing low concentration vitamin D with range 16.8–26.2 ng/mL for Roche; 14–26 ng/mL for Snibe Maglumi, and 9.37–17.4 ng/mL for DiaSorin, and level 2 corresponding to sufficiency range with range of 32.8–49.2 ng/mL for Roche; 35–65 ng/mL for Snibe Maglumi, and 35.2–57.2 ng/mL for DiaSorin. Expanded measurement uncertainty (U) ranged from approximately 9–10% for Maglumi X6, 10–16% for Cobas e801, and up to 20% for Liaison XL, reflecting differences in analytical imprecision and calibration uncertainty.

The lowest Total Error (TE) values were observed for Maglumi X6 (8.43–8.58%), followed by Cobas e801 (9.75–11.69%). In contrast, Liaison XL exhibited substantially higher TE values (19.28–22.65%), primarily driven by higher imprecision and bias.

The results for all instruments compared with the analytical performance specifications (APS), a set of quality requirements against which laboratories can assess the analytical performance of their assays, are shown in Table 5.

## 4. Discussion

The present study demonstrates significant inter-method variability in the measurement of 25-hydroxyvitamin D across three commonly used immunoassays. Although strong correlations were observed between methods, correlation alone does not imply analytical agreement or interchangeability. This distinction has been emphasized in previous studies, which showed that automated immunoassays for 25-hydroxyvitamin D may exhibit high correlation while still presenting significant biases and wide limits of agreement [17,18].

In our study, Bland–Altman analysis confirmed substantial differences between methods, particularly for comparisons involving Cobas e801, where a positive bias was consistently observed for CLIA-based methods Liaison XL and Maglumi X6. In a previous study, we also observed a significant positive bias for a CMIA-based immunoassay (Alinity ci) when compared with the ECLIA-based Cobas e601 [19]. These findings are in line with earlier reports demonstrating that agreement between assays is often method-dependent, with better concordance observed between platforms using similar analytical principles and greater variability when different assay technologies are compared [17,20].

The observed analytical differences translated into clinically relevant discrepancies in patient classification. A considerable proportion of samples were classified depending on the assay used, particularly at the 20 ng/mL threshold for vitamin D sufficiency. The ECLIA instrument (Cobas e801) systematically classified more samples as insufficient, consistent with the negative bias identified in both Passing–Bablok and Bland–Altman analyses. This resulted in high discordance in classification rates exceeding 17% when compared to both Liaison XL and Maglumi X6. In contrast, agreement between the two CLIA-based methods was substantially higher, with minimal reclassification and Cohen’s kappa coefficient exceeding 0.9, indicating near-perfect agreement. At the lower clinical decision threshold (12 ng/mL), agreement was excellent across all platforms, with negligible reclassification, suggesting that inter-method variability has a limited impact at low concentration levels but becomes clinically relevant near decision cut-offs. These findings highlight that even moderate analytical bias can lead to significant clinical misclassification, particularly in the borderline range, and underline the importance of method consistency in patient monitoring and decision-making.

The inter-method variability observed for the Total 25(OH) vitamin D may be partly explained by differences in assay design, including antibody specificity, cross-reactivity with vitamin D metabolites, and the efficiency of 25-hydroxyvitamin D release from its binding protein (VDBP). Lensmeyer et al. demonstrated that 3-epi-25-hydroxyvitamin D_3_, detectable in adult serum samples, evaluated by LC-MS/MS, overestimates total 25(OH) vitamin D by methods unable to chromatographically separate the epimers from vitamin D [21]. In some immunoassays (ECLIA), the cross-reactivity of total 25(OH) vitamin D with 3-epimers was high according to the manufacturers’ claim, but only for the exogenous spiking samples. The study performed by Favresse et al. confirmed the cross-reactivity for both ECLIA vitamin D generations, by using NIST Standard Reference Material 972a enriched with a high concentration of 3-epi-25(OH) vitamin D3, while van den Ouweland et al. reported minimal interferences for endogenous 3-epi- 25(OH) Vitamin D3 [22,23].

In our study, we did not include direct quantification of 3-epi-25(OH)D_3_ or VDBP concentrations in the analyzed samples, nor did we use the LC-MS/MS method for comparability; thus, the actual contribution of these factors to the inter-method discrepancies observed in our cohort cannot be confirmed from our data and remains to be elucidated in dedicated investigations.

Standardization of 25-hydroxyvitamin D measurement remains a major challenge in laboratory medicine, despite ongoing efforts such as the Vitamin D Standardization Program. The VDSP initiative has established performance goals aiming for bias ≤5% and imprecision ≤10%, with the objective of improving comparability between methods and alignment with reference LC-MS/MS procedures.

In the present study, bias estimation based on DEQAS samples demonstrated that, although most platforms showed acceptable agreement with target values, variability persisted between methods. Maglumi X6 and Cobas e801 exhibited similar (−3.6%) and relatively low mean bias compared to CDC-assigned values, while Liaison XL showed an even more pronounced negative bias (−7.6%). Although Cobas e801 and Maglumi X6 exhibited comparable bias, a significant discordance in classification between the two systems was noted at the clinically relevant threshold of 20 ng/mL. These findings are consistent with previous reports indicating that, despite traceability claims to reference materials, immunoassays may still differ in calibration and metabolite recognition [6,18]. The discrepancies observed in this study, particularly those affecting clinical classification, highlight that current standardization efforts have improved but have not eliminated inter-method variability. Considering this, results obtained using different analytical platforms should not be considered interchangeable, especially in clinical decision-making contexts where fixed thresholds are applied.

Based on specifications derived from biological variation, measurement uncertainty (MU) should be less than 13.6% to detect clinically relevant changes and under 9.6% to achieve optimal performance [24]. In this study, only the Maglumi X6 met the threshold for optimal specification, while Cobas e801 showed a concentration dependent variability with MU close to optimal specifications but exceeding the acceptable specification at higher concentrations. Liaison XL exceeded the acceptable limits regardless of concentration levels.

Measurement uncertainty creates the gray zone around the clinically relevant threshold of 20 ng/mL, providing a plausible explanation for the observed reclassification of the patient, depending on the assay used. Cavalier et al. proposed APS for 25 (OH) vitamin D based on MU, proposing values <13.6% as acceptable and <9.6% as optimal [24]; in our study the expanded uncertainty overlapped with the clinical decision threshold of 20 ng/mL yielding a gray zone where small analytical differences may shift patients between different categories, and patients with concentrations near 20 ng/mL are more susceptible to assay-dependent classification changes. This is in line with other reports showing that the proportion of patients classified with vitamin D deficiency can vary depending on the assay [25]. However, this impact on threshold values does not necessarily signify a poor analytical performance of the instruments but rather reflects the limited clinical interchangeability between assays near the clinical decision values, especially linked to the immunoassay response on various vitamin D metabolites. In our study, these analytical differences likely contributed to the systematic bias observed between platforms, particularly the lower concentrations reported by Cobas e801 and the closer agreement between the two CLIA-based methods.

Moreover, the DEQAS results highlighted known limitations of immunoassays in detecting 25(OH) vitamin D_2_, with all methods showing significant deviation from LC-MS/MS assigned values in D_2_-enriched samples. These findings are consistent with previous reports indicating that immunoassay performance may vary depending on metabolite composition and calibration traceability, further contributing to inter-method variability. However, the poor recovery observed in the 25(OH) vitamin D_2_-enriched DEQAS samples should be interpreted in the context of local clinical practice, since in Romania, as in many countries, vitamin D supplementation is exclusively based on 25(OH) vitamin D3. Therefore, the limitations seen in DEQAS 25(OH) vitamin D_2_-enriched samples are expected to have low effect on routine classification in our population. Additionally, the DEQAS report confirmed the presence of 3-epi-25(OH) vitamin D_3_ in evaluated samples, however in low quantity and not specifically exogenous supplemented with this metabolite, without major interferences identified across the three analyzers. So, in contrast to marked variability observed in 25(OH) vitamin D-_2_ enriched samples, the endogenous 3-epi-25(OH) vitamin D did not appear to generate an assay-specific bias pattern. A recent study performed by Shaw and collab. confirmed the manufacturer’s claim, that ECLIA method is the most susceptible at the presence of the exogenous 3-epi-25(OH) vitamin D_3_, compared with other instruments [26]. The presence of physiological 3-epi-25(OH) vitamin D_3_ is abundant in neonates and infants where it may account as a significant circulating vitamin D metabolite, while in adults its concentration is lower, although at detectable levels [27]. All these observations suggest that assay-dependent metabolites may contribute to the variability between methods, influencing the patient classification around the clinical decision thresholds.

Thus, the recognition of method limitations, the differences in calibrator traceability, and metabolite interferences may help in avoiding the misinterpretation near clinical threshold, avoiding systematic shift between assays when multiple methods are used within the same laboratory. At the clinical threshold of severe insufficiency of 12 ng/mL, we found an almost perfect agreement between the studied methods, with minimal assay-dependent reclassification of the patients. Our findings further underline that acceptable analytical comparability between automated immunoassays does not necessarily ensure clinical interchangeability, probably due to differences in metabolite recognition by the antibody of the reagents and assay cross-reactivity, affecting the results and leading to clinically relevant patient reclassification, especially those near clinically relevant thresholds.

In routine clinical laboratory practice, the choice of 25-hydroxyvitamin D assay is frequently dictated by the analytical platforms already in operation. When more than one automated immunoassay platform is concurrently used across different workflows, the results could mislead the diagnostic workflow of the clinician. We have selected these three platforms for comparison because they represent the analytical methods employed in our laboratory for total 25(OH) vitamin D measurement, and because they illustrate three mechanistically distinct immunoassay designs, competitive binding with VDBP-based capture, competitive binding with antibody-based capture, and sandwich-type detection, all of which are currently in widespread clinical use. Our results are in line with those reported by Trimboli et al. who underlined that even widely used immunoassay platforms do not generate interchangeable results, leading to misclassification of the patients [25].

The present study has several limitations. First, no reference method such as LC-MS/MS was included in the comparability analysis, limiting the evaluation to inter-method agreement rather than analytical accuracy. Second, the study was conducted in a single center with a relatively limited sample size, and no stratified analysis by age or biological factors was performed. Third, Bias estimation relied on a limited number of DEQAS samples, which may not fully reflect native patient samples. Finally, potential preanalytical and biological factors, such as vitamin D binding protein, were not assessed.

## 5. Conclusions

The evaluated immunoassays showed generally good overall correlation, however notable inter-method variability was detected, especially near the clinically significant decision threshold of 20 ng/mL. This variability created a ‘gray zone’ where differences between assays led to meaningful discrepancies in patient classification, pointing out that analytical methods for total 25-hydroxyvitamin D are not fully interchangeable. The findings underscore the importance of method consistency in clinical practice and support ongoing efforts toward improved standardization of vitamin D measurement.

## Figures and Tables

**Table 1:** The percentage of samples classified according to the clinically relevant thresholds for deficiency and optimal vitamin D concentration.

Instrument	Median Min–Max ng/mL	<12 ng/mL	≥20 ng/mL
Cobas e801 (*n* = 81)	20.7 (3.87–70.0)	*n* = 5 (6.2%)	*n* = 58 (69.8%)
Liaison XL (*n* = 82)	26.6 (7.93–65.5)	*n* = 4 (4.8%)	*n* = 44 (54.3%)
Maglumi X6 (*n* = 82)	27.7 (8.45–62.8)	*n* = 6 (7.3%)	*n* = 58 (70.7%)

**Table 2:** The kappa coefficient for the strength of the agreement between the three immunoassays at the value of 20 ng/mL, the threshold for insufficiency.

Immunoassay	Kappa Coeff; 95% CI <20 ng/mL	Kappa Coeff; 95% CI <12 ng/mL
Liaison XL vs. Cobas e801	0.642 (0.480–0.804)	1.000 (1.000–1.000)
Maglumi X6 vs. Cobas e801	0.662 (0.501–0.823)	1.000 (1.000–1.000)
Liaison XL vs. Maglumi X6	0.913 (0.816–1.000)	1.000 (1.000–1.000)

Cohen’s kappa coefficient (k) % between the three immunoassays at CDL = clinical decision levels (12 and 20 ng/mL).

**Table 3:** Paired Passing–Bablok regression analysis of the three evaluated methods.

Method	*n*	Correlation Coeff. (95% CI)	Slope (95% CI)	Intercept (95% CI)	Linearity (Cusum)
All samples					
LiaisonXL f(Cobas e801)	81	0.899 (0.847–0.934)	1.145 (1.007–1.300)	0.206 (−2.862; 3.077)	*p* = 0.09
MaglumiX6 f(Cobas e801)	80	0.915 (0.870–0.945)	1.100 (0.987–1.215)	−0.005 (−2.597; 2.347)	*p* = 0.05
LiaisonXL f(Maglumi X6)	82	0.982 (0.973–0.989)	1.042 (1.000–1.087)	0.375 (−0.680; 1.400)	*p* = 0.91
Clinical Decision Interval (10–30 ng/mL)					
LiaisonXL f(Cobas e801)	56	0.787 (0.660–0.870)	1.629 (1.394–2.060)	−8.468 (−16.178; −4.051)	*p* = 0.93
MaglumiX6 f(Cobas e801)	55	0.799 (0.678–0.878)	1.552 (1.316–1.857)	−8.586 (−13.200; −4.084)	*p* = 0.92
LiaisonXL f(Maglumi X6)	46	0.958 (0.924–0.977)	1.099 (0.948–1.184)	0.280 (−2.483; 2.391)	*p* = 0.39

Note: The method (leftmost column) is defined according to the comparator–reference relationship between analyzers, corresponding to the statistical form *y* = *f*(*x*). This ensures that the regression parameters (slope and intercept) are interpreted within the correct directional framework. Accordingly, the equations presented in the table describe the mathematical relationship between the first-listed analyzer (Y) as a function of the second analyzer (X).

**Table 4:** Mean bias (%) calculated across all DEQAS samples (*n* = 10, including one sample enriched with 25(OH) vitamin D_2_), using target value assigned by the CDC VDSP (LC-MS/MS), the All Laboratory Trimmed Mean (ALTM) and the Method Mean (MM).

Instrument	Mean Bias from Target Value (%)	Mean Bias from ALTM (%)	Mean Bias from MM (%)
Cobas e801	−3.6%	0.25%	3.7%
Liaison XL	−7.6%	−4.0%	−0.01%
Maglumi X6	−3.6%	0.3%	−4.0%

Mean bias ALTM = All Laboratory Trimmed Mean; MM = Method Mean.

**Table 5:** The performance specifications for all three instruments and the APS.

Instument	CV	TE	BIAS	MU
Cobas e801	Level 1 = 4.90% Level 2 = 3.73%	Level 1 = 11.69% Level 2 = 9.75%	Level 1 = 3.60% Level 2 = 3.60%	Level 1 = 16.42% Level 2 = 9.82%
Liaison XL	Level 1 = 9.12% Level 2 = 7.08%	Level 1 = 22.65% Level 2 = 19.28%	Level 1 = 7.60% Level 2 = 7.60%	Level 1 = 20.13% Level 2 = 16.52%
Maglumi X6	Level 1 = 3.02% Level 2 = 2.93%	Level 1 = 8.58% Level 2 = 8.43%	Level 1 = 3.60% Level 2 = 3.60%	Level 1 = 9.63% Level 2 = 9.95%
VDSP Requirements	<10%	<15%	<5%	

VDSP = Vitamin D standardization program; CV= coefficient of variation, TE = total error; MU = measurement uncertainty. Level 1 corresponds to low concentration (deficiency) and Level 2 corresponds to high concentration control materials (sufficiency).

## Data Availability

The raw data supporting the conclusions of this article are available as minimal dataset at the publisher.

## Abbreviations

The following abbreviations are used in this manuscript:

CLIA	Chemiluminescent assays
ECLIA	Electro chemiluminescent assay
VDSP	Vitamin D Standardization Program
(ID LC-MS/MS)	Isotope Dilution Liquid Chromatography-Tandem Mass Spectrometry
SRM	Standard Reference Material
CRMs	Certified Reference Materials
25(OH) vitamin D	Total 25-hydroxyvitamin D
SST	Serum separator tubes
RPM	Rotation/minutes
VDBP	Vitamin D binding protein
CLSI	Clinical and Laboratory Standards Institute
AMR	Analytical measurement range
LoD	Limit of detection
LoQ	Limit of quantitation
IFU	Instructions for use
CV	Coefficient of variation
DEQAS	Vitamin D External Quality Assessment Scheme
CDC	Center for Disease Control
ALTM	All Laboratory Trimmed Mean
QC	Quality control
TE	Total error
MU	Measurement uncertainty
APS	Analytical performance specifications

## References

[1] Nardin M., Verdoia M., Nardin S., Cao D., Chiarito M., Kedhi E., Galasso G., Condorelli G., De Luca G. (2024). Vitamin D and Cardiovascular Diseases: From Physiology to Pathophysiology and Outcomes. Biomedicines.

[2] Sha S., Chen L.J., Brenner H., Schöttker B. (2023). Associations of 25-hydroxyvitamin D status and vitamin D supplementation use with mortality due to 18 frequent cancer types in the UK Biobank cohort. Eur. J. Cancer.

[3] Ghaseminejad-Raeini A., Ghaderi A., Sharafi A., Nematollahi-Sani B., Moossavi M., Derakhshani A., Sarab G.A. (2023). Immunomodulatory actions of vitamin D in various immune-related disorders: A comprehensive review. Front. Immunol..

[4] Zhao S.S., Mason A., Gjekmarkaj E., Yanaoka H., Burgess S. (2023). Associations between vitamin D and autoimmune diseases: Mendelian randomization analysis. Semin. Arthritis Rheum..

[5] Wise S.A., Camara J.E., Burdette C.Q., Hahm G., Nalin F., Kuszak A.J., Merkel J., Durazo-Arvizu R.A., Williams E.L., Popp C. (2022). Interlaboratory comparison of 25-hydroxyvitamin D assays: Vitamin D Standardization Program (VDSP) Intercomparison Study 2—Part 2 ligand binding assays—Impact of 25-hydroxyvitamin D2 and 24R,25-dihydroxyvitamin D3 on assay performance. Anal. Bioanal. Chem..

[6] Wise S.A., Kuszak A.J., Camara J.E. (2024). Evolution and impact of Standard Reference Materials (SRMs) for determining vitamin D metabolites. Anal. Bioanal. Chem..

[7] Lee J.H., Seo J.D., Lee K., Roh E.Y., Yun Y.M., Lee Y.W., Cho S.E., Song J. (2024). Multicenter comparison of analytical interferences of 25-OH vitamin D immunoassay and mass spectrometry methods by endogenous interferents and cross-reactivity with 3-epi-25-OH-vitamin D3. Pract. Lab. Med..

[8] Tate J., Ward G. (2004). Interferences in Immunoassay. Clin. Biochem. Rev..

[9] Makris K., Bhattoa H.P., Cavalier E., Phinney K., Sempos C.T., Ulmer C.Z., Vasikaran S.D., Vesper H., Heijboer A.C. (2021). Recommendations on the measurement and the clinical use of vitamin D metabolites and vitamin D binding protein—A position paper from the IFCC Committee on bone metabolism. Clin. Chim. Acta.

[10] Holick M.F., Binkley N.C., Bischoff-Ferrari H.A., Gordon C.M., Hanley D.A., Heaney R.P., Murad M.H., Weaver C.M. (2011). Evaluation, treatment, and prevention of vitamin D deficiency: An endocrine society clinical practice guideline. Med. J. Clin. Endocrinol. Metab..

[11] Liu E.S., Davis A.M., Burnett-Bowie S.A.M. (2025). Vitamin D for Prevention of Disease. JAMA.

[12] Romanian Ministry of Health (2019). Guidelines for Assessing Vitamin D Status in Adults. https://oldsite.ms.ro/wp-content/uploads/2019/07/Anexa2-Ghid-pentru-evaluarea-statusului-vitaminei-D-la-adulti-1-1.pdf.

[13] Cioanta D., Kutos G., Borgovan F., Bene I., Ungur (2025). Impact of Iron, Vitamin D, and B12 Deficiencies on Anemia and Systemic Inflammation: A Retrospective Comparative Study. Rom. J. Lab. Med..

[14] Clinical Laboratory Standard Institute (2018). Measurement Procedure Comparison and Bias Estimation Using Patient Samples EP09-A3. https://clsi.org/.

[15] https://elabdoc-prod.roche.com/eLD/web/ro/ro/documents.

[16] Wise S.A., Camara J.E., Sempos C.T., Lukas P., Le Goff C., Peeters S., Burdette C.Q., Nalin F., Hahm G., Durazo-Arvizu R.A. (2021). Vitamin D Standardization Program (VDSP) intralaboratory study for the assessment of 25-hydroxyvitamin D assay variability and bias. J. Steroid Biochem. Mol. Biol..

[17] Farrell C.J.L., Martin S., McWhinney B., Straub I., Williams P., Herrmann M. (2012). State-of-the-Art Vitamin D Assays: A Comparison of Automated Immunoassays with Liquid Chromatography–Tandem Mass Spectrometry Methods. Clin. Chem..

[18] Carter G.D., Berry J.L., Gunter E., Jones G., Jones J.C., Makin H.L.J., Sufi S., Wheeler M. (2010). Proficiency testing of 25-Hydroxyvitamin D (25-OHD) assays. J. Steroid Biochem. Mol. Biol..

[19] Manescu I.B., Luca A., Hutanu A., Truta A., Dobreanu M. (2024). Anti-thyroid peroxidase (TPO) antibodies—Comparative analysis of two automatic methods, ECLIA and CMIA. Rom. Rev. Lab. Med..

[20] Wallace A.M., Gibson S., de la Hunty A., Lamberg-Allardt C., Ashwell M. (2010). Measurement of 25-hydroxyvitamin D in the clinical laboratory: Current procedures, performance characteristics and limitations. Steroids.

[21] Lensmeyer G., Poquette M., Wiebe D., Binkley N. (2012). The C-3 epimer of 25-hydroxyvitamin D(3) is present in adult serum. J. Clin. Endocrinol. Metab..

[22] Favresse J., Burlacu M.C., Maiter D., Gruson D. (2018). Interferences With Thyroid Function Immunoassays: Clinical Implications and Detection Algorithm. Endocr. Rev..

[23] van den Ouweland J.M.W., Beijers A.M., van Daal H., Elisen M.G.L.M., Steen G., Wielders J.P.M. (2014). Evaluation of 3-epi-25-hydroxyvitamin D_3_ cross-reactivity in the Roche Elecsys Vitamin D Total protein binding assay. Clin. Chem. Lab. Med..

[24] Cavalier E., Fraser C.G., Bhattoa H.P., Heijboer A.C., Makris K., Ulmer C.Z., Vesper H.W., Vasikaran S., Lukas P., Delanaye P. (2021). Analytical Performance Specifications for 25-Hydroxyvitamin D Examinations. Nutrients.

[25] Trimboli F., Rotundo S., Armili S., Mimmi S., Lucia F., Montenegro N., Antico G.C., Cerra A., Gaetano M., Galato F. (2021). Serum 25-hydroxyvitamin D measurement: Comparative evaluation of three automated immunoassays. Pract. Lab. Med..

[26] Shaw A.N., Williams E.L. (2026). 3-Epimer-25-hydroxyvitamin D3 interference in assays for the measurement of 25-hydroxyvitamin D3. J. Steroid Biochem. Mol. Biol..

[27] Singh R.J., Taylor R.L., Reddy G.S., Grebe S.K.G. (2006). C-3 Epimers Can Account for a Significant Proportion of Total Circulating 25-Hydroxyvitamin D in Infants, Complicating Accurate Measurement and Interpretation of Vitamin D Status. J. Clin. Endocrinol. Metab..

